# Effectiveness of the Tier 1 Program of the Project P.A.T.H.S.: Preliminary Objective and Subjective Outcome Evaluation Findings

**DOI:** 10.1100/tsw.2006.238

**Published:** 2006-11-16

**Authors:** Daniel T.L. Shek

**Affiliations:** Quality of Life Centre Hong Kong Institute of Asia-Pacific Studies, Chinese University of Hong Kong, Hong Kong, China

**Keywords:** positive youth development, evaluation, objective outcome evaluation, subjective outcome evaluation, Chinese adolescents

## Abstract

There are two tiers of programs in the Project P.A.T.H.S. (Positive Adolescent Training through Holistic Social Programmes). In the Tier 1 Program, teaching units based on different positive youth development constructs are covered. Pre- and post-test data utilizing the Chinese Positive Youth Development Scale (CPYDS) and post-test subjective outcome evaluation data were collected from 546 students who participated in the 20h Tier 1 Program of the P.A.T.H.S. Project. Results showed that high proportions of the respondents had positive perceptions of the program and the instructors, with 85.3% of the respondents regarding the program as helpful to them. Positive changes in the program participants in many measures of positive youth development were also observed. Although there were some increases in problem behavior in some areas, adolescent problem behavior was generally stable. The present study provides preliminary support for the effectiveness of the Tier 1 Program of the Project P.A.T.H.S. in Hong Kong.

## INTRODUCTION

A survey of the literature shows that there are worrying trends and phenomena related to the development of adolescents in Hong Kong, such as mental health problems, abuse of psychotropic substances, adolescent suicide, school violence, and drop in family solidarity[1,2,3]. As such, primary prevention programs targeting specific adolescent developmental problems and positive youth development programs are called for. However, research findings show that there are very few systematic and multiyear positive youth development programs in Hong Kong. Even if such programs exist, they commonly deal with isolated problems and issues in adolescent development (i.e., deficits-oriented programs), and they are relatively short-term in nature. In addition, systematic and long-term evaluation of the available programs does not exist.

To promote holistic development among adolescents in Hong Kong, the Hong Kong Jockey Club Charities Trust has approved HK$400 million to launch a project entitled “P.A.T.H.S. to Adulthood: A Jockey Club Youth Enhancement Scheme”. The word “P.A.T.H.S.” denotes **P**ositive **A**dolescent **T**raining through **H**olistic **S**ocial Programmes. The Trust has invited academics of five universities in Hong Kong to form a Research Team, with the Chinese University of Hong Kong as the leading institution and the author as the Principal Investigator, to develop a multiyear universal positive youth development program to promote holistic adolescent development in Hong Kong.

Besides developing the program, the Research Team will provide training for teachers and social workers who implement the program and carry out longitudinal evaluation of the project. Different sectors and government departments are also involved in the project. The Social Welfare Department has been engaged as the major partner responsible for the overall coordination and implementation of the project through the assistance of the nongovernmental organizations (NGOs). The Education and Manpower Bureau (EMB) as a collaborator would facilitate the implementation of the project in secondary schools. NGOs operating school social work services may, in consultation with the secondary schools concerned, submit funding proposals to implement the project in the schools they are serving.

There are two tiers of programs (Tier 1 and Tier 2) in this project. The Tier 1 Program is a universal positive youth development program wherein students in Secondary 1 to Secondary 3 will participate, normally with 20h of training in the school year at each grade. Because research findings suggest that roughly one-fifth of adolescents would need help of a deeper nature, a Tier 2 Program will generally be provided for at least one-fifth of the students who have greater psychosocial needs at each grade (i.e., selective program).

In a review of the effectiveness of 77 positive youth development programs, Catalano and his associates[4] concluded that only 25 programs were successful and different positive youth development constructs were incorporated in the successful programs. These constructs include: promotion of bonding, cultivation of resilience, promotion of social competence, promotion of emotional competence, promotion of cognitive competence, promotion of behavioral competence, promotion of moral competence, cultivation of self-determination, promotion of spirituality, development of self-efficacy, development of a clear and positive identity, promotion of beliefs in the future, provision of recognition for positive behavior, provision of opportunities for prosocial involvement, and fostering prosocial norms. To help adolescents to develop in a holistic manner, these 15 adolescent developmental constructs are covered in the project, particularly in the Tier 1 Program.

There are two implementation phases in this project: Experimental Implementation and Full Implementation. For the Experimental Implementation Phase (January 2006 to August 2006), 52 secondary schools were invited to participate in the project with the objectives of accumulating experiences in program implementation and familiarizing front-line workers with the program design and philosophy. In 2006/07 school year, the programs will be implemented on a full scale at Secondary 1 level. In 2007/08 school year, the programs will be implemented at Secondary 1 and Secondary 2 levels. In 2008/09 school year, the programs will be implemented at Secondary 1, Secondary 2, and Secondary 3 levels.

The project is pioneering and groundbreaking in Hong Kong and other Chinese contexts in many aspects. First, this is the first positive youth development project that provides 60h of universal training on positive youth development in the junior secondary school years in Hong Kong. Second, it includes both universal and selective programs (i.e., primary and secondary prevention programs). Third, it includes the development of a multiyear positive youth development program, provision of training on a massive scale, and implementation of rigorous evaluation. Fourth, it involves the collaboration of five universities, different government departments, NGOs providing school social work services, and the education sector. Fifth, it utilizes longitudinal evaluation over a long period of time involving the collection of different data from different sources.

Although much resource has been injected to program development, staff training, and program implementation, one fundamental question that should be asked is whether the program is effective in helping the program participants in positive youth development. In the Western context, the review of

Catalano et al.[4] showed that among the 77 programs under review, only roughly one-third of them was effective. In the context of Hong Kong, Shek et al.[5] concluded that evidence-based social work practice was very weak and there is a need to accumulate research findings on the effectiveness of psychosocial intervention programs. Against this background, this study attempts to provide some evidence on the effectiveness of the Tier 1 Program indexed by changes in the participants after joining the Tier 1 Program in the Experimental Implementation Phase.

Adopting the criteria based on positivism and post-positivism, Biglan et al.[6] suggested that there are different levels of evidence that researchers and practitioners can consider. For Grade 1 evidence, Grade 2 evidence is met and the preventive intervention is implemented in its intended setting with sufficient staff training and monitoring of implementation and outcomes. For Grade 2 evidence, two or more independent research teams are involved in multiple, well-designed, randomized, controlled trials or multiple, well-designed, interrupted, time-series experiments. For Grade 3 evidence, a single research team is involved in multiple, well-designed, randomized, controlled trials or multiple, well-designed, interrupted, time-series experiments. For Grade 4 evidence, it refers to at least one well-designed, randomized, controlled trial or an interrupted, time-series design that is replicated across three cases. For Grade 5 evidence, it refers to evidence based on nonequivalent group design (i.e., participants are not randomly assigned to the experimental group and control group). For Grade 6 evidence, it refers to the use of pre-experimental design without the involvement of a control group. Finally, Grade 7 evidence refers to clinical experience by respected researchers and practitioners and case reports.

Ideally, randomized group trials in multiple sites conducted by different teams of independent researchers should be attempted. However, there are several practical difficulties involved. First, it is very expensive to conduct randomized group trials in different settings. Second, it is difficult to assign schools and clients to the control group (e.g., some schools may refuse to join the control group). For example, McCall et al.[7] reviewed three major summative approaches to evaluation (randomized experimental designs, nonequivalent control group designs, and interrupted time-series designs) and remarked that "methodologically, it is now acknowledged that conducting robust true experiments in the field – the scientific ideal described at the beginning of this chapter – is extremely difficult and often impossible" (p. 982). Third, randomized controlled trials may not be the best strategy for positive youth development programs at the earlier stage of a program. Finally, for researchers not upholding the tenets of positivism or post-positivism, a randomized control trial may not be regarded as a superior form of strategy that can yield knowledge about the program effect.

As the Project P.A.T.H.S. is at its beginning stage in Hong Kong, an evaluation based on the one-group pre- and post-test design in the Experimental Implementation Phase was carried out. Although there are limitations of evaluation study based on the one group pre- and post-test design, this strategy has been widely used in human services evaluation research. Thyer[8] pointed out that there are many myths in program evaluation (e.g., "you must control for the most relevant threats to internal validity" and "you must randomly assign clients to various control and experimental groups") and suggested that pre-experimental designs (e.g., one group pre- and post-test design) are valuable strategies that could be meaningfully used in practice setting. Finally, subjective outcome evaluation data were collected in this study to look at the program effect from an alternative perspective.

## METHODS

### Participants and Procedures

Among the 52 schools joining the Experimental Implementation Phase, there were 29 schools adopting the full program (i.e., 20h program involving 40 units). To allow for a longer duration of the program implementation, 14 schools implemented the program before the official implementation date (i.e., January 1, 2006). Among the 7 schools that began the implementation of the program in November to December 2005, 4 schools were randomly selected to participate in this study. There were 656 and 652

students who participated in the pre-test and post-test, respectively. Discarding those questionnaires that showed doubtful responses (19 questionnaires at pre-tests and 22 questionnaires at post-test), usable questionnaires at pre- and post-tests could be successfully matched for 546 students.

At pre- and post-tests, the purpose of the study was mentioned and the confidentiality of the data collected was repeatedly emphasized to all of the students in attendance on the day of testing. The students were asked to indicate their wish if they did not want to participate in the study at the time of administration at each wave of data collection (i.e., "passive" informed consent was obtained from the students). All participants responded to all scales in the questionnaire in a self-administration format. Adequate time was provided for the participants to complete the questionnaire.

## Instruments

At pre- and post-tests, the participants were invited to respond to a questionnaire including measures of positive youth development, thriving, life satisfaction, adolescent problem behavior, and demographic information. The measures focused in this paper are described below.

## The Chinese Positive Youth Development Scale (CPYDS)

Regarding the assessment of positive development of the program participants, the Chinese Positive Youth Development Scale (CPYDS) was used. The CPYDS was found to possess adequate psychometric properties[9]. Based on reliability analyses of both the pre- and post-test data in the present study, there were some slight modifications in the composition of the items of the 15 subscales of the CPYDS are as follows:

1. Resilience Subscale (6 items): $\alpha = 0.83$ and 0.82 at pre- and post-tests
2. Social Competence Subscale (7 items): $\alpha = 0.80$ and 0.86 at pre- and post-tests
3. Emotional Competence Subscale (6 items): $\alpha = 0.83$ and 0.86 at pre- and post-tests
4. Cognitive Competence Subscale (6 items): $\alpha = 0.85$ and 0.87 at pre- and post-tests
5. Behavioral Competence Subscale (6 items): $\alpha = 0.70$ and 0.62 at pre- and post-tests
6. Moral Competence Subscale (6 items): $\alpha = 0.76$ and 0.78 at pre- and post-tests
7. Self-Determination Subscale (modified 4 items): $\alpha = 0.82$ and 0.82 at pre- and post-tests
8. Self-Efficiency Subscale (modified 2 items): $\alpha = 0.48$ and 0.54 at pre- and post-tests
9. Beliefs in the Future Subscale (modified 2 items): $\alpha = 0.70$ and 0.66 at pre- and post-tests
10. Clear and Positive Identity Subscale (7 items): $\alpha = 0.84$ and 0.86 at pre- and post-tests
11. Spirituality Subscale (7 items): $\alpha = 0.85$ and 0.88 at pre- and post-tests
12. Bonding Subscale (6 items): $\alpha = 0.83$ and 0.85 at pre- and post-tests
13. Prosocial Involvement Subscale (5 items): $\alpha = 0.80$ and 0.86 at pre- and post-tests
14. Prosocial Norms Subscale (5 items): $\alpha = 0.73$ and 0.78 at pre- and post-tests
15. Recognition for Positive Behavior Subscale (4 items): $\alpha = 0.77$ and 0.79 at pre- and post-tests

Several composite indices based on the above measures were also formed. According to Shek et al.[9], the total score based on all 15 subscales could be used as an overall measure of positive development. However, as it can be argued that constructs including spirituality, prosocial norms, prosocial involvement, bonding, and recognition for positive behavior are different from the rest of the scales, a summation of 10 subscales assessing psychosocial competence and strengths could be used (i.e., resilience, social competence, emotional competence, cognitive competence, behavioral competence, moral competence, self-determination, self-efficiency, beliefs in the future, and clear and positive identity). Finally, different types of competencies (i.e., social, emotional, cognitive, behavioral, and moral competencies) were combined to form the domain of psychosocial competence. It would be expected that if the Tier 1 Program was effective in promoting positive youth development, the post-test scores would be significantly higher than the pre-test scores on the related measures.

### Subjective Outcome Evaluation Scale

Twenty items were used to assess the participants satisfaction with the course and instructor as well as their perceived benefits of the program at post-test. Reliability analysis showed that this measure was reliable ($\alpha = 0.97$).

### Measures of Adolescent Developmental Problems

Eight items were used to assess the participant's frequency of using alcohol, tobacco, "ice" (methylamphetamine), cannabis, cough mixture, organic solvent, tranquilizers, and narcotics[10]. In addition, 12 items were used to assess the frequency of delinquent behavior in the previous year, including stealing, cheating, truancy, running away from home, damaging others' properties, assault, having sexual relationship with others, gang fighting, speaking foul language, staying away from home without parental consent, strong-arming others, and breaking into others' places[10]. Finally, five items were used to assess the adolescents' behavioral intention to engage in problem behavior, including drinking, smoking, taking drugs, having sex with others, and participation in gambling[11].

## RESULTS

Regarding the participants' perceptions of the course, instructor, and benefits of the Tier 1 Program, the percentage findings in Table 1 revealed several observations. First, 82% of the participants perceived that the program could help them to reflect. Second, roughly 85% of the respondents were appreciative of the encouragement given by the instructor. Third, a high proportion of the participants (85.3%) regarded the program as helpful to them.

The findings also revealed that there were positive changes in the program participants (Table 2). The program participants generally showed improvement with reference to the composite measures of positive youth development based on 10 domains and psychosocial competence, respectively. With specific reference to those participants who joined the Tier 1 Program only and regarded the program was helpful to them (N = 341), there were positive changes in: (a) the composite measure involving 10 domains of positive youth development, (b) the composite measure on psychosocial competence, and (c) some of the subscales. Some examples of the positive changes at the item level are also presented.

Results also showed that there was generally no deterioration of adolescent developmental problems in substance abuse ($p > 0.05$ in 7 items), delinquency ($p > 0.05$ in 11 items), and behavioral intention to engage in problem behavior ($p > 0.05$ in 4 items). However, there were increases in drinking, deceiving others, speaking foul language, and behavioral intention to drink alcohol from pre- to post-tests (Table 2).

## DISCUSSION

The findings based on the subjective outcome evaluation strategy or client satisfaction survey showed that a very high proportion of the respondents had positive perceptions of the program and the instructor. Most importantly, roughly 85% of the respondents regarded the program as helpful to them. The use of subjective outcome indicators or the client satisfaction approach in evaluation has a long history in human services in different cultures[12]. Although there are arguments against the use of subjective outcome assessment[13], there is evidence showing that subjective outcome measures were correlated with objective outcome measures[14]. The subjective outcome evaluation findings in this study provide support for the positive program effects of the Tier 1 Program of the P.A.T.H.S. Project.

**TABLE 1**
**Findings Based on the Subjective Outcome Evaluation Strategy**

| | | Percentage of Responses (%) | | | | | | | |
|---|---|---|---|---|---|---|---|---|---|
| | | **1** | **2** | **3** | **A** | **4** | **5** | **6** | **B** |
| 1. | The atmosphere of the class was good. | 3.9 | 6.3 | 10.8 | **21.0** | 33.8 | 30.3 | 14.9 | **79.0** |
| 2. | There were many opportunities for students to exchange ideas during class. | 2.6 | 4.6 | 8.8 | **16.0** | 34.6 | 35.1 | 14.3 | **84.0** |
| 3. | I often had encouragement from the responses of classmates. | 2.2 | 5.9 | 9.4 | **17.5** | 33.9 | 35.9 | 12.7 | **82.5** |
| 4. | I think students actively participated in the class activities. | 2.2 | 4.1 | 10.5 | **16.8** | 32.6 | 34.8 | 15.8 | **83.2** |
| 5. | I think I actively participated in the class activities. | 1.7 | 4.2 | 9.2 | **15.1** | 31.7 | 36.7 | 16.4 | **84.8** |
| 6. | I think the discipline in class was good. | 4.4 | 7.0 | 13.8 | **25.2** | 31.6 | 29.4 | 13.8 | **74.8** |
| 7. | I think this course was very interesting. | 2.8 | 4.8 | 12.3 | **19.9** | 30.4 | 31.1 | 18.6 | **80.1** |
| 8. | I think this course encouraged me to reflect. | 1.8 | 4.8 | 11.4 | **18.0** | 29.6 | 36.2 | 16.2 | **82.0** |
| 9. | I like this course very much. | 2.8 | 5.5 | 10.5 | **18.8** | 29.0 | 32.2 | 20.0 | **81.2** |
| 10. | The instructor could arouse my interest in the course. | 2.6 | 5.0 | 11.0 | **18.6** | 28.5 | 35.7 | 17.1 | **81.3** |
| 11. | The instructor could arouse my learning motivation. | 2.8 | 4.0 | 10.7 | **17.5** | 30.1 | 35.1 | 17.3 | **82.5** |
| 12. | The instructor knew how to promote discussion and participation among the students. | 1.7 | 3.7 | 10.9 | **16.3** | 29.3 | 35.4 | 19.2 | **83.9** |
| 13. | I could get encouragement from the responses of the instructor. | 1.3 | 5.0 | 9.2 | **15.5** | 31.1 | 34.3 | 19.2 | **84.6** |
| 14. | I often share the things I have learned from the course with my friends. | 3.5 | 6.8 | 15.1 | **25.4** | 28.5 | 31.3 | 14.9 | **74.7** |
| 15. | I often share the things I have learned from the course with my family members. | 6.8 | 6.8 | 16.8 | **30.4** | 31.3 | 25.2 | 13.1 | **69.6** |
| 16. | I think this course could strengthen my ability to face the challenge of life. | 2.8 | 4.3 | 10.2 | **17.3** | 30.7 | 36.8 | 15.3 | **82.8** |
| 17. | I think this course could strengthen my ability to face adversity. | 2.6 | 4.6 | 11.9 | **19.1** | 29.8 | 34.6 | 16.5 | **80.9** |
| 18. | I think this course could increase my understanding about myself. | 3.3 | 4.1 | 9.2 | **16.6** | 29.2 | 37.8 | 16.4 | **83.4** |
| 19. | I think this course could promote my overall development. | 3.5 | 3.7 | 8.3 | **15.5** | 28.9 | 36.3 | 19.3 | **84.5** |
| 20. | Overall speaking, I think this course was helpful to me. | 3.7 | 3.3 | 7.7 | **14.7** | 26.5 | 36.4 | 22.4 | **85.3** |

*Note*: 1 = Strongly Disagree, 2 = Disagree, 3 = Slightly Disagree, 4 = Slightly Agree, 5 = Agree, 6 = Strongly Agree, A = Sum of the disagree responses (i.e., negative responses), B = Sum of the agree responses (i.e., positive responses).

Regarding the objective outcome evaluation, the findings are generally positive and they are consistent with the original expectations. In particular, significant differences between pre- and post-tests were found in terms of the composite measure based on 10 subscales and the composite measure of psychosocial competence. Obviously, the program effect could be regarded as encouraging in view of the short duration of the intervention program.

**TABLE 2**
**Changes in the Program Participants Based on the Different Measures of Positive Youth Development and Adolescent Developmental Problems**

| Variables/Subscales | Pre-Test | | Post-Test | | t Value |
|---|---|---|---|---|---|
| | Mean | SD | Mean | SD | |
| **All Participants (N = 546)** | | | | | |
| Total Scale | 4.46 | 0.64 | 4.48 | 0.64 | –0.66n |
| Personal Development Score | 4.37 | 0.70 | 4.44 | 0.68 | –2.95b |
| Psychosocial Competence Score | 4.41 | 0.70 | 4.51 | 0.68 | –3.43b |
| **Participants Who Regarded the Program as Effective (N = 464)** | | | | | |
| Total Scale | 4.53 | 0.61 | 4.58 | 0.57 | –2.06a |
| Personal Development Score | 4.42 | 0.67 | 4.54 | 0.59 | –4.60c |
| Psychosocial Competence Score | 4.48 | 0.68 | 4.61 | 0.60 | –4.72c |
| **Participants Who Joined the Tier 1 Program Only and Regarded the Program as Effective (N = 341)** | | | | | |
| Total Scale | 4.59 | 0.58 | 4.60 | 0.55 | –0.49n |
| Personal Development Score | 4.47 | 0.65 | 4.56 | 0.57 | –3.24b |
| Psychosocial Competence Score | 4.51 | 0.66 | 4.62 | 0.59 | –3.39b |
| Resilience Subscale | 4.74 | 0.77 | 4.81 | 0.68 | –1.87d |
| Emotional Competence Subscale | 4.25 | 0.91 | 4.45 | 0.84 | –4.25c |
| Cognitive Competence Subscale | 4.48 | 0.83 | 4.71 | 0.71 | –4.89c |
| Self-Determination Subscale | 4.71 | 0.85 | 4.80 | 0.72 | –2.12a |
| Clear and Positive Identity Subscale | 4.21 | 0.87 | 4.30 | 0.85 | –2.01a |
| Recognition for Positive Behavior (Item 2) | 4.49 | 1.04 | 4.60 | 0.96 | –1.78d |
| Moral Competence (Item 6) | 4.17 | 1.23 | 4.39 | 1.09 | –3.05b |
| Beliefs in the Future (Item 1) | 4.40 | 1.17 | 4.53 | 0.97 | –2.08a |
| **Adolescent Problem Behavior** | | | | | |
| Alcohol consumption (e.g., beer) | 1.47 | 0.99 | 1.63 | 1.12 | –3.17b |
| Delinquency (deceiving others) | 2.16 | 1.61 | 2.50 | 1.83 | –3.25b |
| Delinquency (speaking foul language) | 2.64 | 1.99 | 2.90 | 2.21 | –2.49a |
| Behavioral intention (drinking alcohol) | 1.68 | 0.99 | 1.82 | 1.04 | –2.60a |

*Note*: Total Scale = mean of the all subscales in the CPYDS. Personal Development Score = mean of 10 subscales excluding Bonding, Prosocial Involvement, Prosocial Norms, Spirituality, and Recognition for Positive Behavior subscales. Psychosocial Competence Score = mean of social, emotional, cognitive, behavioral, and moral competencies subscales. Some examples showing positive changes at the item level are presented. Recognition for Positive Behavior (Item 2): "When I help my classmates, my behavior is recognized by them." Moral Competence (Item 6): "I have the habit of self-evaluation." Beliefs in the Future (Item 1): "I have confidence to solve my future problem."

a $p < 0.05$ (two-tailed)
b $p < 0.01$ (two-tailed)
c $p < 0.001$ (two-tailed)
d $p < 0.05$ (one-tailed)
n Non-significant

Nevertheless, no significant differences were observed for the more socially related domains, such as bonding, prosocial norms, and prosocial involvement. Of course, it can be argued that as development in such areas may take a longer time to be nurtured, the short duration of the intervention may not be able to reflect such changes adequately. In addition, as the process of individuation in early adolescence actually increases the distance between the adolescents and their parents and other healthy adults, the expected effect of the program in this area may not be prominent in this case.

Finally, it is noteworthy that most of the adolescent problem behavior remained stable. This observation can be considered as a good sign because there are many studies showing that adolescent delinquent behavior and substance abuse problems increased over time, particularly in the adolescent years[10]. In some sense, the lack of significant differences between pre- and post-tests in terms of most of the measures of adolescent problem can be interpreted as the beneficial program effect. Of course, the significant increases in drinking, deceiving others, speaking foul language, and intention to drink alcohol still deserve our attention. The finding on adolescent drinking is consistent with the view of Lou and Shek[15] that adolescent drinking is an emergent problem in Hong Kong. Obviously, comparison data based on a control group must be collected to give further insight on the effectiveness of the intervention program.

As the design of the study is a pre-experimental design, there are different alternative explanations for the positive changes observed. One possible alternative explanation is that the positive changes in the program participants were due to maturation effect. However, this explanation can be partially dismissed as the positive changes did not appear in those who did not regard the program to be helpful. In addition, the alternative explanation that the program success was due to the benefits of other programs can be partially dismissed as none of the schools in this study participated in the major youth enhancement programs in Hong Kong, including the Adolescent Health Project and the Understanding the Adolescent Project. In conjunction with the qualitative evaluation data reported elsewhere[16], it can be concluded that there is positive evidence supporting the effectiveness of the Tier 1 Program of the P.A.T.H.S. Project[16].

## ACKNOWLEDGMENTS

The preparation for this paper and the Project P.A.T.H.S. were financially supported by the Hong Kong Jockey Club Charities Trust.
